# ASO Author Reflections: Intraductal Papillary Mucinous Neoplasm-Derived Pancreatic Cancer: A Need to Evaluate for Specific Staging Systems

**DOI:** 10.1245/s10434-024-16131-w

**Published:** 2024-09-10

**Authors:** Joseph R. Habib, Ingmar F. Rompen, Ammar A. Javed, Lois A. Daamen

**Affiliations:** 1 Department of Surgery, New York University Langone Health, New York, NY USA; 2 Department of Surgery, Regional Academic Cancer Center Utrecht, UMC Utrecht Cancer Center and St. Antonius Hospital Nieuwegein, Utrecht, The Netherlands; 3 Department of Surgery, Amsterdam UMC, University of Amsterdam, Amsterdam, The Netherlands; 4 Cancer Center Amsterdam, Amsterdam, The Netherlands; 5 Division of Imaging and Oncology, University Medical Center Utrecht, Utrecht, The Netherlands; 6 Department of Surgery, Regional Academic Cancer Center Utrecht, Utrecht, The Netherlands

## Past

Traditionally, intraductal papillary mucinous neoplasm (IPMN)-derived pancreatic cancer has been staged, treated, and surveilled in a similar manner to the more common pancreatic intraepithelial neoplasm (PanIN)-derived pancreatic cancer.

## Present

Emerging evidence suggests that IPMN-derived pancreatic cancers are biologically and clinically distinct entities;^1^ however, due to a lack of robust literature, IPMN-derived pancreatic cancer is still managed like PanIN-derived pancreatic cancer. As a result, there is an urgent need to reinvestigate the currently implemented prognostic, treatment, and surveillance strategies in this unique patient population. In particular, when considering staging of disease, smaller studies have suggested that the currently employed American Joint Committee on Cancer (AJCC) 8th edition system is inaccurate. Expert consensus guidelines have recommended T1 substaging for IPMN-derived pancreatic cancer, given the observation that it is often resected at smaller tumor sizes.^2^ Accordingly, unlike in PanIN-derived pancreatic cancers, it was recently reported that a T1 substaging is valid and prognostic for IPMN-derived pancreatic cancer.^3^ Current literature recommends reversion to the binary (positive or negative) AJCC 7th edition for nodal staging in IPMN-derived pancreatic cancers. However, this large, international, multicenter study found that multiple cut-offs of nodal positivity, including the currently employed AJCC 8th edition for N staging, perform well in stratifying patients based for recurrence-free and overall survival.^4^ In light of this, an adequate lymphadenectomy of at least 10 lymph nodes is required to prevent inaccurate nodal staging.^5^

## Future

Although some management and staging may overlap with PanIN-derived pancreatic cancer, there is an urgent need for more robust evidence to support these guidelines and evaluate their applicability in IPMN-derived pancreatic cancer. It is already apparent that some deviations exist, and thus specific IPMN-derived pancreatic cancer staging and guidelines are likely warranted.
